# Development of an engineered peptide antagonist against periostin to overcome doxorubicin resistance in breast cancer

**DOI:** 10.1186/s12885-020-07761-w

**Published:** 2021-01-14

**Authors:** Khine Kyaw Oo, Thanpawee Kamolhan, Anish Soni, Suyanee Thongchot, Chalermchai Mitrpant, Pornchai O-charoenrat, Chanitra Thuwajit, Peti Thuwajit

**Affiliations:** 1 Graduate Program in Immunology, Department of Immunology, Faculty of Medicine Siriraj Hospital, Mahidol University, Bangkok, 10700 Thailand; 2 Department of Immunology, Faculty of Medicine Siriraj Hospital, Mahidol University, Bangkok, 10700 Thailand; 3 Bachelor of Science Program in Biological Science (Biomedical Science), Mahidol University International College, Mahidol University, Nakhon Pathom, 73170 Thailand; 4 Siriraj Center of Research Excellence for Cancer Immunotherapy (SiCORE-CIT), Faculty of Medicine Siriraj Hospital, Mahidol University, Bangkok, 10700 Thailand; 5 Department of Biochemistry, Faculty of Medicine Siriraj Hospital, Mahidol University, Bangkok, 10700 Thailand; 6 Department of Surgery, Faculty of Medicine Siriraj Hospital, Mahidol University, Bangkok, 10700 Thailand; 7 Breast Center, Medpark Hospital, Bangkok, 10110 Thailand

**Keywords:** Breast cancer, Periostin, Chemoresistance, Phage display 12-amino acids library, Doxorubicin

## Abstract

**Background:**

Chemoresistance is one of the main problems in treatment of cancer. Periostin (PN) is a stromal protein which is mostly secreted from cancer associated fibroblasts in the tumor microenvironment and can promote cancer progression including cell survival, metastasis, and chemoresistance. The main objective of this study was to develop an anti-PN peptide from the bacteriophage library to overcome PN effects in breast cancer (BCA) cells.

**Methods:**

A twelve amino acids bacteriophage display library was used for biopanning against the PN active site. A selected clone was sequenced and analyzed for peptide primary structure. A peptide was synthesized and tested for the binding affinity to PN. PN effects including a proliferation, migration and a drug sensitivity test were performed using PN overexpression BCA cells or PN treatment and inhibited by an anti-PN peptide. An intracellular signaling mechanism of inhibition was studied by western blot analysis. Lastly, PN expressions in BCA patients were analyzed along with clinical data.

**Results:**

The results showed that a candidate anti-PN peptide was synthesized and showed affinity binding to PN. PN could increase proliferation and migration of BCA cells and these effects could be inhibited by an anti-PN peptide. There was significant resistance to doxorubicin in PN-overexpressed BCA cells and this effect could be reversed by an anti-PN peptide in associations with phosphorylation of AKT and expression of survivin. In BCA patients, serum PN showed a correlation with tissue PN expression but there was no significant correlation with clinical data.

**Conclusions:**

This finding supports that anti-PN peptide is expected to be used in the development of peptide therapy to reduce PN-induced chemoresistance in BCA.

**Supplementary Information:**

The online version contains supplementary material available at 10.1186/s12885-020-07761-w.

## Background

Breast cancer (BCA) is one of the global public health problems. According to the International Agency for Research in Cancer Fact Sheet, 2018 showed that BCA in approximately 2,088,849 people year, was in the top rank of new cancer cases worldwide [1]. Regularly, the mortality of cancer is attributed to various processes in cancer progression, including metastasis, proliferation and chemoresistance. These processes were influenced by not only the properties of cancer cells themselves but also to the effects of the tumor microenvironment (TME). TME is therefore now considered a therapeutic target [2]. Periostin (PN) is one of active molecules in TME that has been reported to be a promoter of cancer progression through various mechanisms including proliferation, invasion/migration, angiogenesis and chemoresistance [3, 4]. PN expression is up-regulated in the TME of many types of cancer, for example, non-small cell lung cancer (NSCLC) [5], malignant pleural mesothelioma [6], prostate cancer [7], renal cell carcinoma [8], cholangiocarcinoma (CCA) [9] and also BCA [10]. Regularly, PN molecules are secreted from cancer-associated fibroblasts (CAF) and can activate the cancer cells via integrin receptors [3]. PN expression in cancer tissue was significantly correlated to survival of patients in colorectal cancer (CRC) [11], CCA [9] and BCA [12]. In addition, serum PN was also positively correlated with poor prognosis in CRC [13] and CCA [14]. In BCA, however, the correlation between patient survival and serum PN is still controversial since no correlation [15] or strong correlation [16] have been reported. PN is also related to chemoresistance of cancer cells, usually via PI3K/Akt/survivin signaling to increase cell viability in the presence of chemotherapeutic agents [17, 18]. Chemoresistance is one of the main difficulties for cancer treatment that can lead to mortality of the patients, therefore, the understanding and manipulation of chemoresistance can support patient survival.

PN has been reported to be associated with many kinds of chemotherapeutic drugs, such as arsenic trioxide in hepatocellular carcinoma (HCC) [19]; methotrexate, doxorubicin [20], cisplatin [21], carboplatin and paclitaxel [22] in ovarian cancer (OVC); oxaliplatin and 5-fluorouracil (5-FU) [18] in CRC and gemcitabine in pancreatic cancer [23]. Moreover, PN was also shown to be associated with anti-angiogenic therapy [24]. It was found that inhibition of PN can restore the sensitivity to chemotherapeutic drugs [25]. Since, almost cytotoxic drugs have serious adverse effects that can lead to morbidity and mortality [26], a minimum of drug usage should be of benefit to cancer patients. To overcome chemoresistance from PN, a bioactive peptide is one of the interesting molecules to develop an inhibitory mechanism [27]. A bioactive peptide can be defined as a peptide that can bind to a molecular target and has an effect on cells or organisms. It has advantageous properties for use in cancer therapy such as high tissue penetration as compared to full size antibodies, good biocompatibility and binding affinity to target molecules [27]. There are many peptides used for therapeutic purposes including cancer treatment [28]. To date, the database web site http://crdd.osdd.net/raghava/thpdb/ [29] reported 61 peptides approved by United States Food and Drug Administration (US-FDA) for cancer therapy [30]. Peptides can be used as a high-throughput screen for many methods including phage biopanning from the peptide library [27].

In this study, a 12 amino acid peptide library was used for screening of bioactive peptides that could bind to PN at an integrin binding site [31] and inhibit PN function of BCA cells in in vitro experiments. In addition, screening of PN expression in cancer tissue and measurement if serum PN levels from BCA patients were performed and the correlation analyzed with clinical elements including the response to anthracycline-based chemotherapy. The application of this peptide may be used in future clinical practice to restore the sensitivity to chemotherapeutic drugs in BCA cells and reduce the dosages in patients which could decrease the morbidity and mortality from the adverse effects of the drug.

## Methods

### Cell cultures of BCA cells

BCA cell lines, MDA-MB-231 and MCF-7 were used in the study. They were cultured with Dulbecco Modified Eagle’s Medium (DMEM) (Gibco, Thermo Fisher Scientific, Waltham, MA, USA). These media contained 10% fetal bovine serum (FBS) (Gibco), using penicillin/streptomycin (Gibco) as antibiotics and amphotericin B (Gibco) as an antifungal drug with 5% CO_2_ and 90% humidity at 37 °C.

Lipofectamine™ 3000 (Invitrogen, Thermo Fisher Scientific) was used to transfect the blank pCDNA™3.1 plasmid (v385–20, Invitrogen) or pCDNA™3.1 PN-plasmid into BCA cell lines. After transfection, the cells were selected by Geneticin™ (Gibco) (up to 1 mg/ml) to create stable cell lines. PN and integrin expressions were tested by reverse transcriptase (RT)-polymerase chain reaction (PCR) using Light Cycler® 480 II system (Roche, Basel, Switzerland) with specific primers (Table 1) [9, 32]. Glyceraldehyde-3-phosphate dehydrogenase (*GAPDH*) mRNA expression was used as an internal control (Table S1). The cycle threshold (Ct) value was used for calculation of expression folding.

### Western blot analysis

To determine the secreted PN amount in conditioned-media of each cell, 3 × 10^5^ cells were seeded into 6-well plates with 3 ml of complete media. Cell media were removed the next day and washed with phosphate-buffered saline (PBS), and serum free media was added. Media was collected at 24 h, centrifuged and supernatant was taken and concentrated by Vivaspin®6 (VS0691, Sartorius, Goettingen, Germany). Protein concentration was determined by Bradford reagent (#5000006, Bio-Rad Laboratories, Hercules, CA, USA) and the amounts were adjusted to 5 μg per loading. To determine protein expression or phosphorylation in the cellular part, 2 × 10^6^ cells were lysed with RIPA buffer (sc-24,948, Santa Cruz Biotechnology, Inc., Dallas, TX, USA). Each sample was separated by sodium dodecyl sulfate (SDS) polyacrylamide gel electrophoresis (PAGE) and transferred to PVDF membrane. The membranes were blocked with 5% bovine serum albumin (BSA) and immunodetection for PN was continued with goat anti-PN polyclonal antibody (sc49480, Santa Cruz Biotechnology) and rabbit anti-goat IgG conjugated with horseradish peroxidase (HRP) (HAF017, R&D Systems, Minneapolis, MN, USA**)** for PN detection, rabbit anti-Akt polyclonal antibody (#9272, Cell Signaling Technology, Danvers, MA, USA), rabbit anti-pAkt polyclonal antibody (#9271, Cell Signaling Technology), rabbit anti-survivin polyclonal antibody (#2803, Cell Signaling Technology) and goat anti-rabbit-HRP (ab6721, Abcam, Cambridge, UK) for AKT, phosphorylated AKT (pAKT) and survivin. β-actin expression was determined as an internal control for the cellular part using mouse anti-β-actin polyclonal antibody (sc47778, Santa Cruz Biotechnology) and horse anti-mouse-HRP (#7076, Cell Signaling Technology). HRP was detected by Pierce™ enhanced chemiluminescence (ECL) reagent (Thermo Fisher Scientific) and chemiluminescence signal was detected by G:BOX gel documentation system (Syngene, Cambridge, UK). Expression level of PN was determined by ImageJ version 1.52a software (National Institutes of Health, Bethesda, MD, USA).

### Immunocytochemistry

BCA cells, approximately 1 × 10^5^ cells, were plated on top of coverslips in 24-well plates and cultured for 24 h. After that, media was removed and cells were washed with PBS, fixed with 4% paraformaldehyde, washed again and blocked with 5% FBS. The primary antibody used was mouse anti-human integrin αVβ5 monoclonal antibody (MAB2019Z, Sigma-Aldrich, Merck KGaA, Darmstadt, Germany), at a concentration of 15 μg/ml and incubated with the cells at 37 °C for 4 h. After that, cells were washed with PBS and incubated with Cy™3 AffiniPure F (ab’)_2_ Fragment Goat Anti-Mouse IgG, Fcγ fragment specific (115–166-071, Jackson Immuno Research Inc., West Grove, PA, USA), at a concentration of 0.5 μg/ml, at room temperature (RT) for 1 h. Hoechst 33258 was used for nuclear staining. Confocal imaging experiments were conducted on a Zeiss LSM 800 (Carl Zeiss, Jena, Germany) at the Division of Molecular Medicine, Faculty of Medicine Siriraj Hospital, Mahidol University. Equipment details were microscope model: AxioObserver7, objective lens: Plan-Apochromat 63x/1.4NA oil immersion and laser: Diode 561 nm. Acquisition software was ZEN 2.3 software (blue edition, 2002–2011).

### Serum and tissue specimens

Serum was obtained from left-over specimens of pre-operative BCA patients and normal females who came for health checks for determination of PN concentrations. Cancer tissues from BCA patients were taken from the remaining tissue after pathological examination. Serum and tissues and clinical information collections were performed by Prof. Pornchai O-charoenrat, Department of Surgery, Faculty of Medicine Siriraj Hospital, Mahidol University, under agreement of Siriraj Institutional Review Board No. Si519/2010.

### Phage biopanning

Ph.D.™-12 phage display peptide library (New England Biolabs, Ipswich, MA, USA) was used for phage biopanning of anti-PN peptide following the manufacturer’s protocol. In summary, a peptide fragment correlated to the integrin binding site of PN [31] conjugated with biotin (Biotin-ERIMGDKVASEALMKYHILN) was added into Pierce™ Streptavidin Coated High Capacity Plates (#15500, Thermo Fisher Scientific). Continuously, 10^10^ plaque forming units (pfu) of bacteriophage were poured into the wells and incubated at RT for 1 h. Unbound phages were then washed off and bound phages were collected. Phage titering was continued using *E. coli* strain ER2738 (New England Biolabs). The bacteria were centrifuged, and supernatant with virus was kept in a fresh tube. Phage precipitation was performed by adding of 1/6 volume of NaCl/polyethylene glycol solution (20% w/v PEG-8000 with 2.5 M NaCl). After that, phage titering was observed on LB/IPTG/Xgal plates, and the amplified phages were used for next round. In this way, the panning process was repeated seven times. During the biopanning process, a negative selection for phage clones was also performed to exclude streptavidin and plastic binding phage. Twenty phage clones per round from third, fifth and seventh rounds were randomly selected for DNA sequencing. Selection of candidate phage clones was done according to the results of sequencing. The sequence with highest frequency was assumed as the best phage clone to be used for further experiments. The sequences were also checked by online database to target unrelated peptides (http://i.uestc.edu.cn/sarotup3/index.html) [33] and to identify and rule out the peptide sequences which had high probability of binding to streptavidin and plastic more than 0.5. The binding affinity of selected phage clones were confirmed by the dot blot method. Volumes of 1 μl with 500 ng of recombinant PN (rPN) (RD172045025, BioVendor, Brno, Czech Republic) or BSA were spotted on nitrocellulose membranes, dried for 15 min and placed in 96-well plates then blocked with 5% BSA. Membranes were incubated with the selected phage or blank phage clones (10^12^ pfu in 50 μl) at 4 °C overnight. Then membranes were washed and incubated with 50 μl (2 μg/ml) of anti-M13 antibody-HRP (ab50370, Abcam) at RT for 1 h and detected by ECL.

### Peptide design and synthesis

After selecting the best binding sequence of 12-amino acids peptides, 2 types of peptide would be synthesized, plain peptide and peptide conjugated with fluorescein isothiocyanate (FITC). For the synthesis of the latter, a spacer region (GGGSCK) would be added at the C-terminal end of the peptide and FITC was conjugated with the side chain of lysine. Finally, C-terminal amidation would be performed. The synthesis of plain and FITC-labelled anti PN peptides was ordered from Syn Peptide company (Shanghai, China). FITC-labelled anti-PN peptide tested binding affinity to non-denaturing cell lysate of transfected BCA cells and their mock transfected cells and rPN by dot blot analysis. Briefly, 12.5 μg of cell lysate or 500 ng of rPN in 1 μl was applied onto nitrocellulose membrane. The membrane was blocked with 5% BSA followed by peptide incubation at 4 °C overnight and the fluorescent signal was detected the next day using the G:BOX gel documentation system. The checking of anti-PN peptide binding to intact PN-transfected BCA cells was also performed with similar process as immunocytochemistry plus a step of cell membrane permeabilizing after fixation by incubated with 1% Triton X for 1 min at RT. The single staining step was done by incubation of the permeabilized cells with 2 μM FITC-labelled anti-PN peptide for 1 h at RT, washed and then nuclear stained with Hoechst 33258. The observation was viewed under confocal microscope using laser diode 488 nm.

### Determination of peptide binding affinity

The physical properties of this peptide would be determined by an online tool (https://www.thermofisher.com) (Thermo Fisher Scientific). Structure of this peptide and the binding properties to PN would be predicted by PEP-FOLD3 [34] and pepATTRACT [35] tools from RPBS Web Portal (https://bioserv.rpbs.univ-paris-diderot.fr) using PN 3D-structure from RCSB PDB database (https://www.rcsb.org/structure/5YJG) [36]. Binding affinity of anti-PN peptide was determined with isothermal titration calorimetry [37] using the MicroCal PEAQ-ITC Machine (Malvern Panalytical Ltd., Malvern, UK) in which 700 nM of rPN (or BSA as negative control) and either 70 nM of commercial goat anti-PN polyclonal antibody or 100 nM of plain anti-PN peptide in 50 mM Tris and 150 mM NaCl (pH 7.5) buffer were added into syringe and cell compartments of the machine. The procedure followed the instructions for the machine. The results would be determined by the measurement of the exothermic energy after intermittent injection into the cell compartment and reported as the binding affinity constant (KD).

### Proliferation assay

The proliferation assay of BCA cells was determined by the cell viability assay using CellTiter 96® Aqueous One Solution Cell Proliferation Assay (MTS assay) (Promega, Madison, WI, USA). Briefly, 3 × 10^3^ cells in 100 μl medium were seeded in 96-well plates for 24 h. The day 1 baseline determination used the MTS assay following company instructions. For the experiment, cells were placed into new media containing 2% FBS with or without rPN (100 ng/ml) and anti-PN peptide (1 μM) and cells were cultured for a further 72 h. After treatment, cell viability was measured by the MTS assay. Proliferation rate was calculated as the folding of cell numbers increased from the baseline.

### Determination of cell stemness in PN-transfected cell and effect of anti-PN peptide

Stemness of PN-transfected BCA cells was determined by staining with anti-CD24 and anti-CD44 antibodies and analyzed by flow cytometry and compared with mock-transfected cells in conditions without or with anti-PN peptide. Briefly, BCA cells were seeded at the concentration 1 × 10^5^ cells into 6-well plates with 2 ml of complete media. Cell media was removed the next day and changed to 1% FBS media for 24 h. Then, the treatment in 1% FBS media without or with anti-PN peptide (1 μM) were refilled and incubated for a further 24 h. At the end of treatment, cell pellets were collected and incubated in 2% FBS/1x PBS with 1:20 dilution of FITC-labeled anti-CD24 antibody (cat no. 21270043, ImmunoTools GmbH, Friesoythe, Germany) and 1:5 dilution of allophycocyanin (APC)-labeled anti-CD44 antibody (cat no. 21270446, ImmunoTools) for 30 min. CytoFLEX® Flow cytometry (Beckman Coulter, Inc. Brea, CA, USA) and CytExpert® software version 2.1 (Beckman Coulter, Inc.) were used for analysis.

### Determination of the half-maximal inhibitory concentration (IC50)

Determination the IC50 of BCA cells in response to chemotherapeutic drugs was analyzed by MTS assay. The experiment started from 5 × 10^3^ of BCA cells in 100 μl medium that were seeded in 96-well plates for 24 h, then media was changed with different concentrations of new chemotherapeutic drugs [doxorubicin (S1208), paclitaxel (S1150), cisplatin (S1166), 5-fluorouracil (5-FU) (S1209) and gemcitabine (S1714) that were purchased from Selleck Chemicals, Houston, TX, USA] with or without rPN and anti-PN peptide for a further 48 h. After that, cell viability was determined by the MTS assay. Combination index (CI) was calculated by IC50 of combination of drug and PN or anti-PN peptide divided by IC50 of chemotherapeutic drug alone.

### Migration assay

The wound healing assay was performed to determine migration activity of the BCA cells after treatment under various conditions. Briefly, 5 × 10^4^ cells of parental BCA cells or mock/PN-transfected cells were seeded in 24-well culture plates with their regular media for 24 h and media changed to 1% FBS with or without rPN (100 ng/ml) and anti-PN peptide (1 μM) and culture continued for 1 day until they reached approximately 95% confluency. Wounds were applied by scratching using 200 μl micropipette tip in a single straight line. The media with floating cells would be removed and refilled with the new experimental media. The culture process would be continued for a further 24 h and photos of the area would be taken at 8 h intervals. The migration area would be analyzed from the photos taken using TScratch version 1.0 software (https://github.com/cselab/TScratch) [38] and migration activity calculated as μm^2^/h. Independent duplicated experiments were performed.

### Enzyme-linked immunosorbent assay (ELISA) for serum PN measurement

Periostin ELISA Kit (Human) (Shino-Test Corporation, Tokyo, Japan) was used for measurement of serum PN following the procedure as previously described [14].

### Immunohistochemistry

Matching tissues with the serum PN measurement from BCA patients were used for PN staining. An immuno-peroxidase staining procedure was performed using the method in paraffin-embedded BCA tissues as previously described. The area and intensity of expression was estimated and semi-quantitatively graded as 0–3 scores. For area determination, 0 was up to 5%, 1 was 6–25%, 2 was 26–50% and 3 was 51–100% of either the cancer or fibroblast area. For intensity scoring, 0 was negative, 1 was weakly positive, 2 was moderately positive and 3 was strongly positive when compared to positive and negative controls. Quick score (Q-score) was determined by multiplying the area and intensity scores. For statistical analysis, the scores of 0–4 were categorized as low expression, and 6–9 as high expression.

### Statistical analysis

ANOVA test or Student’s *t*-test were applied for statistical analysis of the experiments. GraphPad Prism® version 7.04 software (GraphPad Software, San Diego, CA, USA) was used in calculation of IC50 values of chemotherapeutic drugs and PASW Statistics software version 18 (SPSS, IBM, Armonk, NY, USA) was used for other analysis. Dose-responses were compared by Holm-Sidak’s multiple comparison test. Mann-Whitney ranked-sum test was used for comparisons between normal and patient serum PN. Chi-square or Fisher’s exact test were used to determine the correlation between PN expression and clinical data in BCA patients. Kaplan-Meier Log-rank test using Kaplan-Meier Plotter online tool (https://kmplot.com/) was performed to determine the correlation between PN expression and survival time of BCA patients by online database [39]. *P*-values of < 0.05 were used as statistical significance.

## Results

### Expression of integrins in BCA cell lines and PN transfection

BCA cells were used to check the expressions of integrin α5, α6, αV, β1, β 3, β 4 and β5 by real time RT-PCR and all cells showed detection of signal amplification (Fig. S1). In addition, integrin αVβ5 heterodimer was detected by fluorescence immunocytochemical staining. The fluorescence signal was presented in all BCA cells with a membrane pattern (Fig. S2). Successful PN transfection in BCA cell lines were tested using real time RT-PCR and western blot analysis of condition medium. Real time RT-PCR of *PN* determined up-regulation in MDA-MB-231 and MCF-7 as approximately 1 × 10^2^ and 5 × 10^3^ fold compared with mock control cells (Fig. S3a). PN detection in conditioned medium of both PN-transfected BCA cell lines showed increased PN when compared with their mock control cells (Fig. S3b).

### Anti-PN peptide screening, production and binding affinity test

**Fig. 1:**
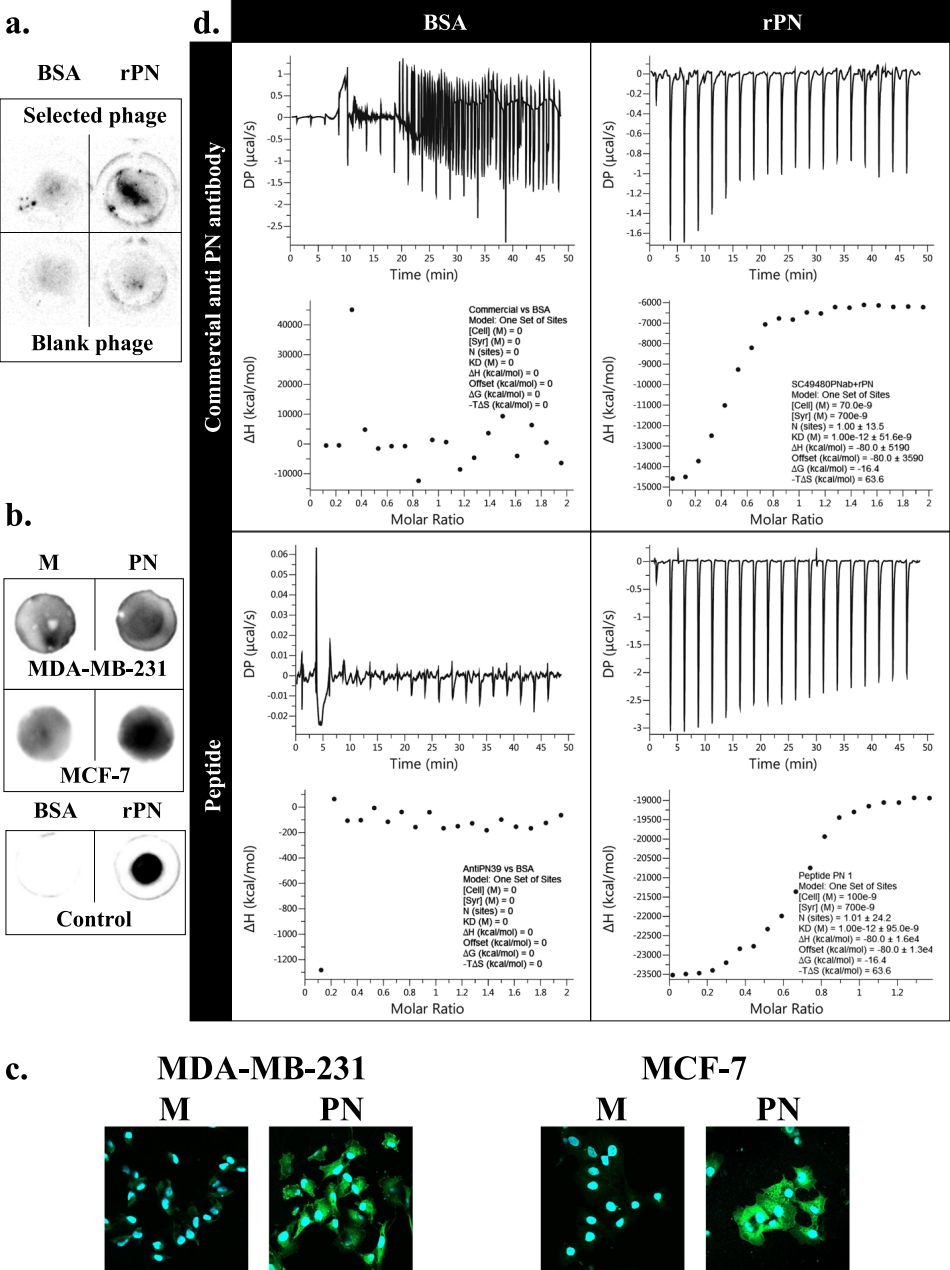
Binding of anti-PN peptide to PN. **a** Phage binding to rPN, compared with blank phage and BSA was used as the negative control, **b** Peptide binding to non-denaturing cell lysate from PN-transfected BCA cells compared with mock transfected BCA cells and BSA/ was used rPN as negative/positive control (M = mock transfected and PN = PN-transfected), **c** Peptide binding to permeabilized PN-transfected BCA cells compared with mock transfected BCA cells, visualized by confocal microscope with 640X original magnification, nucleus was adjusted to similar brightness, and (**d**) Isothermal titration calorimetry analysis diagram from binding between anti-PN peptide and BSA or rPN compared with commercial anti-PN antibody

Phage-biopanning was used for screening of anti-PN peptide. After performing of 7-rounds of phage-biopanning, a candidate peptide with a sequence of TFATHGKHWAAP was selected. This clone presented 1 copy in the fourth round and 3 copies in the seventh round and had binding scores to streptavidin and plastic as 0.22 and 0.19. The physical properties of this peptide were determined (Fig. S4). Peptide structure was determined as linear with small alpha helix (Fig. S5a) and binding to PN active site had been predicted with binding energy − 11.89 kCal/mol (Fig. S5b). Phage binding to rPN using dot blot analysis showed a higher signal compared with a blank phage (Fig. 1a). After that, 2 types of anti-PN peptide were synthesized, plain anti-PN peptide and FITC-labelled anti-PN peptide. FITC-labelled anti-PN peptide was tested for binding to rPN and non-denaturing cell lysate PN-transfected BCA cells. The results determined the binding of anti-PN peptide higher in both PN-transfected BCA cells compared with their mock control cells and were markedly increased in rPN but had no signal in BSA (Fig. 1b). This was confirmed by immunocytochemistry staining in permeabilized cells (Fig. 1c). Binding affinity of anti-PN peptide to rPN was also determined by isothermal titration calorimetry using commercial goat anti-PN polyclonal antibody as a positive control. These results indicated that anti-PN peptide also showed acceptable binding affinity to rPN (KD = 1 pM) similar to commercial antibody, but could not be determined in BSA (Fig. 1d).

### Effects of anti-PN peptide on cell proliferation and migration

**Fig. 2:**
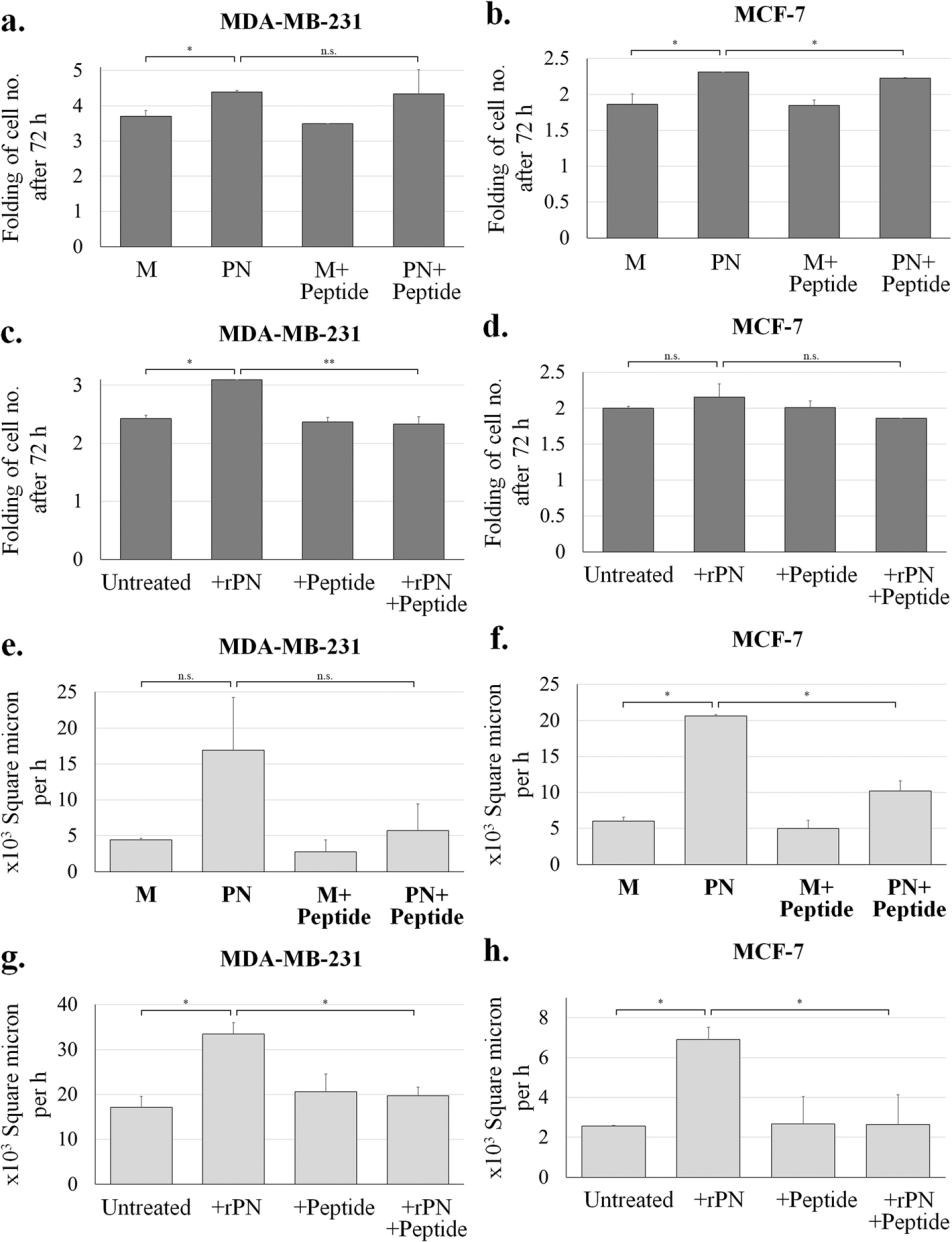
Proliferation and migration of PN and inhibition by anti-PN peptide. **a-b** Proliferation effect by the PN-transfected experiment compared with mock transfected BCA cells without or with anti-PN peptide, **c-d** Proliferation effect study by rPN and/or anti-PN peptide treatment, **e-f** Migration study by wound healing assay compared between PN and mock transfected BCA cells and inhibition by anti-PN peptide and (**g-h**) Migration study by wound healing assay compared between by rPN treatment BCA cells and inhibition by anti-PN peptide. M = mock transfected, PN = PN-transfected, error bar determined standard error of mean (SEM), * = *P*-value < 0.05, ** = *P*-value < 0.001 and n.s. = not significant

From the cell proliferation assay in the PN transfection experiment, PN-transfected cells showed increased cell number within 72 h higher than the mock transfected control with statistical significance (*P*-value = 0.029 and 0.049 for MDA-MB-231 and MCF-7 cells) (Fig. 2a-b). Anti-PN peptide could significantly reduce cell proliferation only in PN-transfected MCF-7 cells (*P*-value = 0.006) (Fig. 2b), but not in other mock and PN-transfected cells (Fig. 2a-b). In the PN treatment experiment, the 72-h treatment significantly showed induction of cell proliferation by the PN treatment in MDA-MB-231 (*P*-value = 0.004) (Fig. 2c). In addition, anti-PN peptide could significantly counteract PN in MDA-MB-231 cells (*P*-value < 0.001) (Fig. 2c). For MCF-7, PN could increase cell numbers and anti-PN peptide could decrease this effect but did not show significance statistically (Fig. 2d).

The cell migration assay by PN transfection experiment was performed for 24 h with 8 h intervals for image capture. The analysis was done in appropriate interval because at 24 h some showed completely closed wounds. The results showed that PN-transfected cells had a higher migration rate than the mock transfected control with statistical significance for MCF-7 (*P*-values = 0.002) but was not significant in MDA-MB-231cells (Fig. 2e-f). Anti-PN peptide significantly reduced cell migration in PN-transfected MCF-7 cells (*P*-value = 0.018) but was not significant in MDA-MB-231 cells (Fig. 2e-f). In the PN treatment experiment, the results showed that rPN-treated cells had a higher migration rate than the untreated control with statistical significance for both MDA-MB-231 and MCF-7 (*P*-values = 0.033 and 0.030 for MDA-MB-231 and MCF-7 cells) (Fig. 2g-h). Anti-PN peptide also significantly reduced cell migration in both rPN-treated MDA-MB-231 and MCF-7 cells (*P*-value = 0.026 and 0.035) (Fig. 2g-h).

### Effect of anti-PN peptide on drug resistance

PN-transfected BCA cell lines were used to determine the IC50 of chemotherapeutic agents compared with their mock controls. Only doxorubicin showed the increased IC50 in both PN-transfected BCA cells with statistical significance (for MDA-MB-231 cell, IC50 of PN-transfected cell/mock transfected cell = 1.6 with *P*-value = 0.032 and for MCF-7 cell, IC50 of PN-transfected cell/mock transfected cell = 1.6 with *P*-value = 0.035) (Fig. S6a). PN-transfected MCF-7 in paclitaxel test tests showed significant increases of IC50 (IC50 of PN-transfected cell/mock transfected cell = 1.5 with *P*-value = 0.044) (Fig. S6c). Therefore, the next experiments with anti-PN peptide was performed using doxorubicin.

**Fig. 3:**
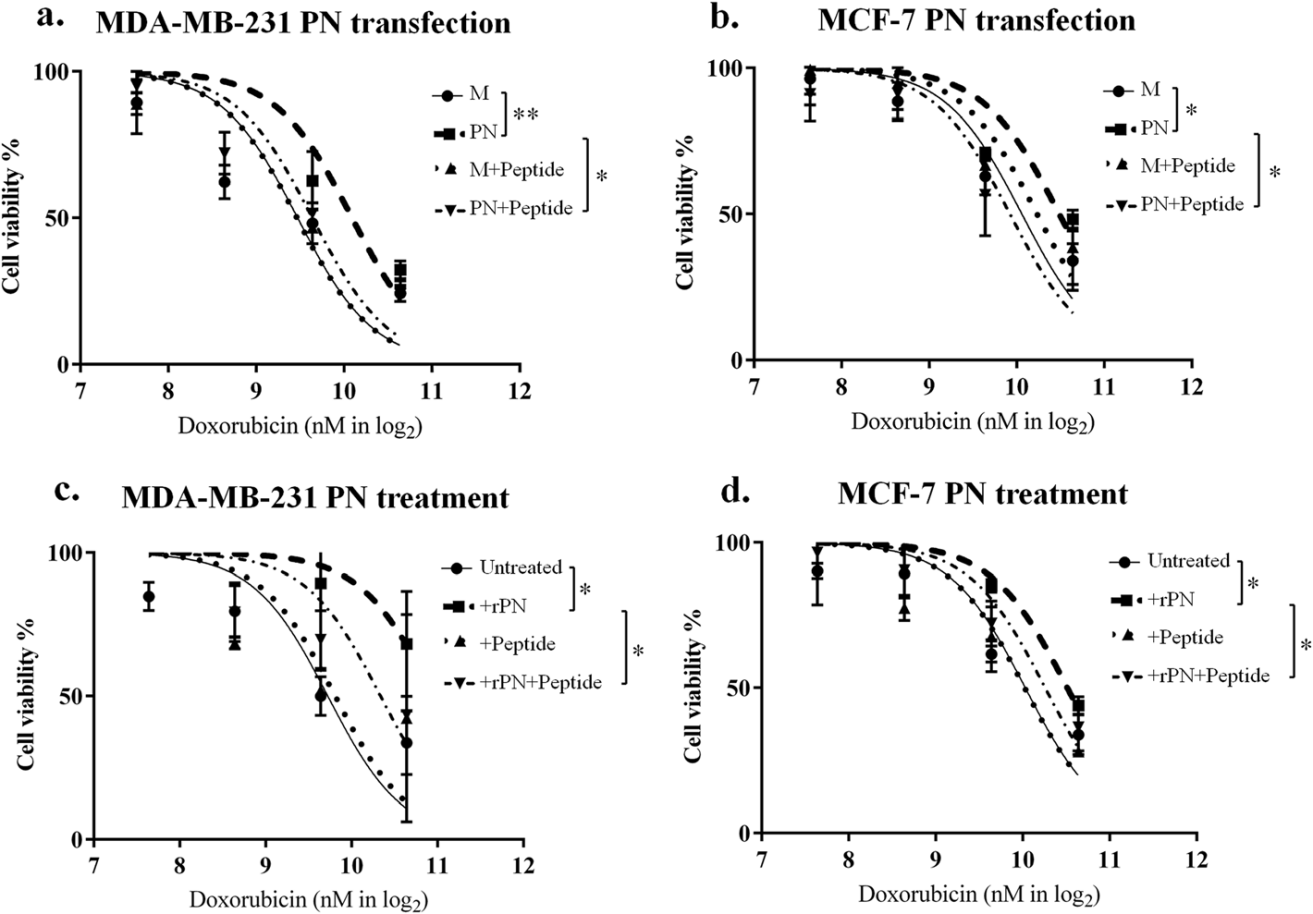
Dose-response curve of doxorubicin. **a-b** PN-transfection experiment. **c-d** rPN treatment experiment. M = mock transfected, PN = PN-transfected, error bar determined SEM, * = *P*-value < 0.05 and ** = *P*-value < 0.001

The experiment for testing the ability of anti-PN peptide to reverse the drug resistance effect of PN was performed using doxorubicin treatment. The experiments were performed in both PN-transfected BCA cells and with PN treatment. For PN transfection, a dose-response curve of both PN-transfected BCA cells were shifted to the right with significant *P*-values (for MDA-MB-231 cell, IC50 of mock transfected cell = 709.67 nM and PN-transfected cell = 1089.92 nM with *P*-value < 0.001 and for MCF-7 cell, IC50 of mock transfected cell = 1067.48 nM and PN-transfected cell = 1438.15 nM with *P*-value = 0.013) and showed that anti-PN peptide could significantly shift values to the left (for MDA-MB-231 cell, IC50 of PN-transfected cell with anti-PN peptide = 797.31 nM with *P*-value = 0.002 and for MCF-7 cell, IC50 of PN-transfected cell with anti-PN peptide = 970.11 nM with *P*-value = 0.007) with minimal effect on mock transfected cells (for MDA-MB-231 cell, IC50 of mock transfected cell with anti-PN peptide = 705.74 nM with *P*-value = 0.932 and for MCF-7 cell, IC50 of PN-transfected cell with anti-PN peptide = 1200.98 nM with *P*-value = 0.376) (Fig. 3a-b). The CI of anti-PN peptide in doxorubicin treated PN-transfected MDA-MB-231 cell was 0.73 and of PN-transfected MCF-7 cell was 0.68.

For PN treatment experiment, dose-response curves of both PN-transfected BCA cells were also shifted to the right with significant *P*-values (for MDA-MB-231 cell, IC50 of untreated control cell = 844.53 nM and rPN-treated cell = 1992.00 nM with *P*-value = 0.024 and for MCF-7 cell, IC50 of untreated control cell = 1045.52 nM and rPN-treated cell = 1458.23 nM with *P*-value = 0.007) and anti-PN peptide could be significantly shifted back to the left (for MDA-MB-231 cell, IC50 of rPN + anti-PN peptide treated cell = 1296.13 nM with *P*-value = 0.038 and for MCF-7 cell, IC50 of rPN + anti-PN peptide treated cell = 1266.22 nM with *P*-value = 0.038) with minimal effect on condition without rPN (for MDA-MB-231 cell, IC50 of anti-PN peptide treated cell = 896.40 nM with *P*-value = 0.870 and for MCF-7 cell, IC50 of anti-PN peptide treated cell = 1038.29 nM with *P*-value = 0.558) (Fig. 3c-d). The CI of PN in doxorubicin treated MDA-MB-231 cell was 2.36 and of MCF-7 cell was 1.39. Anti-PN peptide could reverse this with CI of doxorubicin+PN treated MDA-MB-231 cell was 0.65 and MCF-7 cell was 0.89.

### Intracellular pathway corresponded to PN and anti-PN peptide treatment

**Fig. 4:**
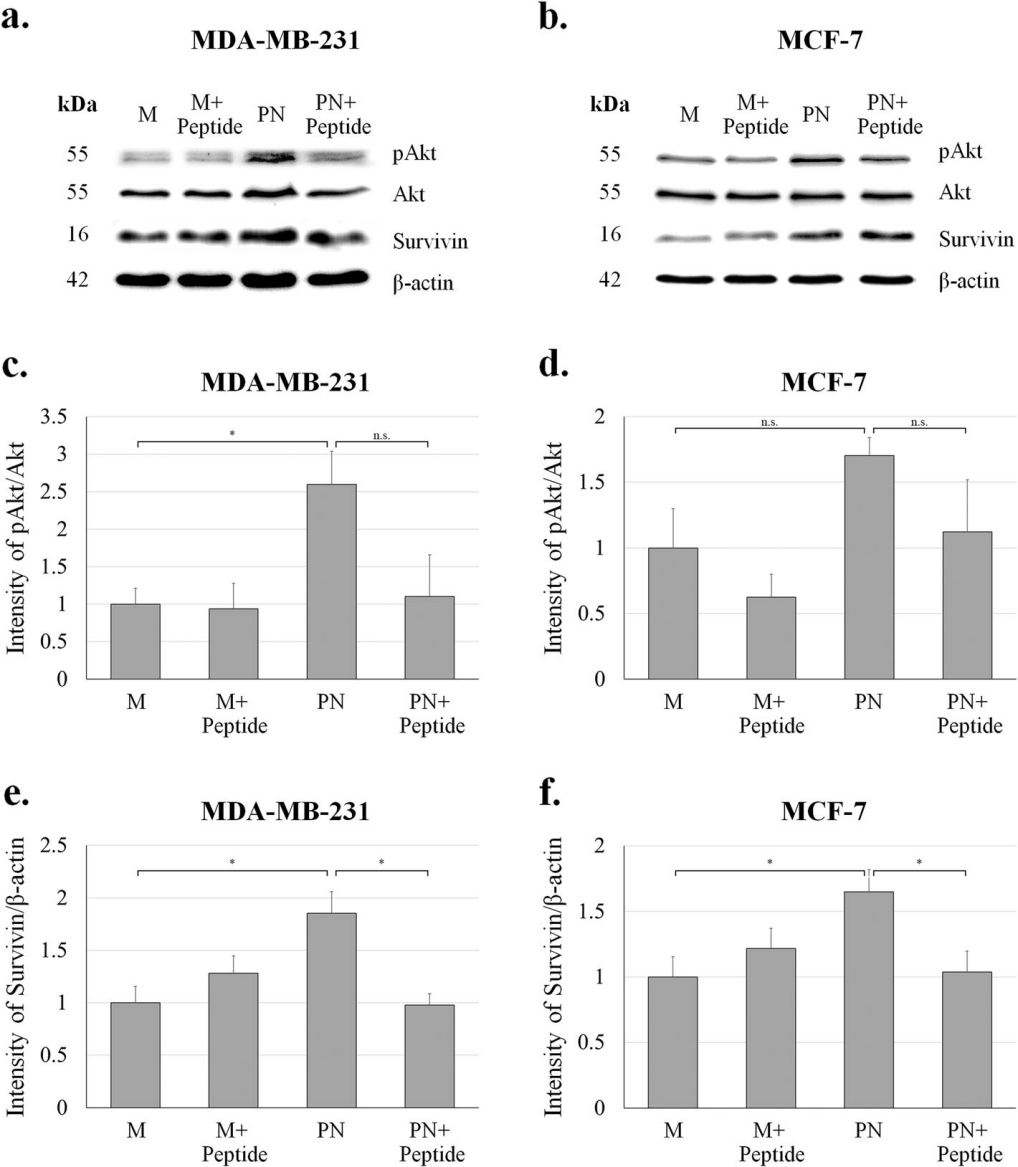
Western blot analysis for expression of pAKT and survivin. **a-b** Signal from immunodetection of pAkt, Akt, survivin and β-actin as the internal control. **c-d** Intensity of pAKT normalized by Akt expression. **e-f** Intensity of survivin normalized by β-actin expression. M = mock transfected, PN = PN-transfected, error bar determined SEM, * = *P*-value < 0.05 and n.s. = not significant

Parental BCA cells were treated with PN and anti-PN peptides and the intracellular signaling pathway determined by western blot analysis. Akt phosphorylation and expression of survivin were detected. From duplicate experiments, averages of band density from image analysis after being normalized with expression of β-actin were plotted and compared. The results determined that PN could increase phosphorylation of Akt and survivin expression in all BCA cells in this experiment and anti-PN peptide could reduce these effects (Fig. 4).

**Fig. 5:**
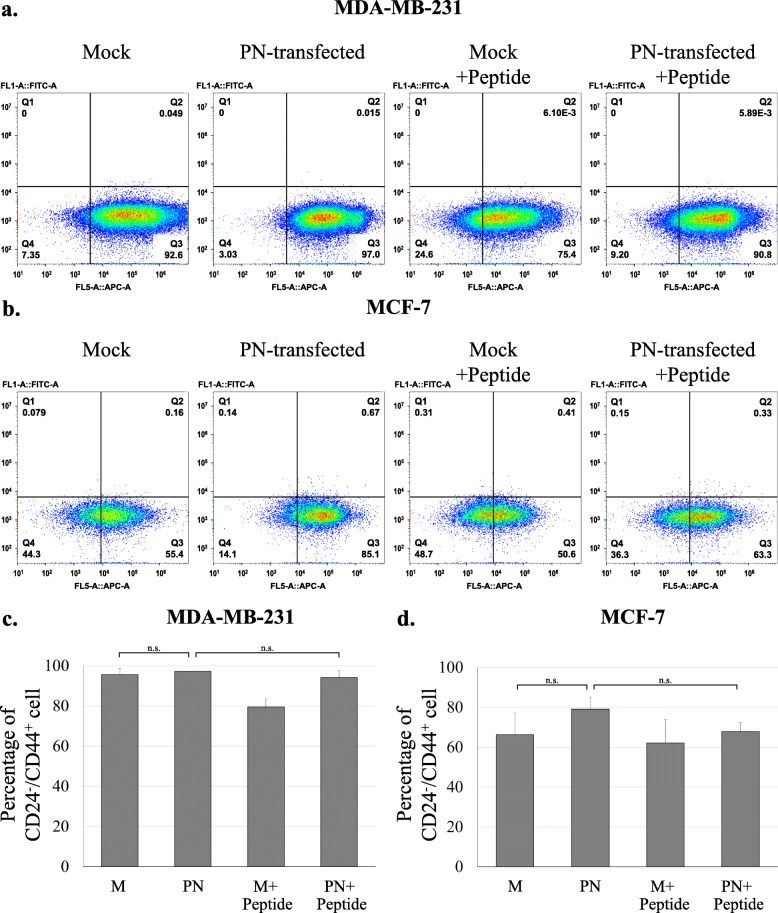
Stemness analysis of PN-transfected cells compared with mock-transfected cells without or with anti-PN peptide treatment. Cells were stained with anti-CD24-FITC and anti-CD44-APC antibodies and cell with CD24^−^/CD44^+^ was marked as cancer stem cell. **a-b** Representative of cytogram from flow cytometer analysis. **c-d** Bar graph of percentage of CD24^−^/CD44^+^ cell in each condition, error bar determined SEM and n.s. = not significant

The determination of stemness as CD24^−^/CD44^+^ cells was shown in Fig. 5. The results indicated that mock-transfected MDA-MB-231 cell contained higher population of stem cells than MCF-7. PN-transfected cell showed minimal increasing of stem cell percentage in MDA-MB-231 cell (95.6 to 97.2, *P*-value = 0.675) and anti-PN peptide could non-significantly reduce stemness of PN-transfected cell (97.2 to 94.2, *P*-value = 0.477). PN-transfected MCF-7 cell showed more raising of CD24^−^/CD44^+^ cell count but not statistical significance (66.3 to 79.2, *P*-value = 0.407) and anti-PN peptide could reduce this effect (79.2 to 67.9, *P*-value = 0.267).

### PN expression and clinical significance in BCA patients

**Fig. 6:**
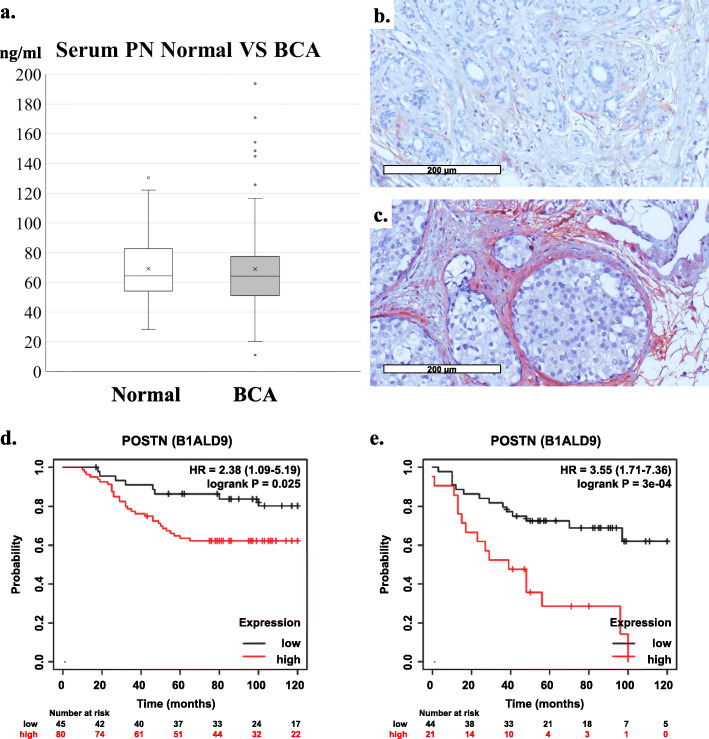
PN expression and clinical significance in BCA patients. **a** Comparison of serum PN levels between BCA patients and normal controls. BCA patients *n* = 83, normal controls *n* = 60. **b-c** Tissue expression of PN in BCA tissue. PN staining presented only in stromal area but not cancer cells. **b** Low expression of PN, Q-score = 1 and (**c**) High expression of PN in patient with highest serum PN level, Q-score = 6. Original magnification was 200X. Scale bar represented 200 μm length. **d-e** Survival analysis of high PN expression compared with low PN expression in BCA patients from online database analyzed by Kaplan-Meier Plotter online tool (https://kmplot.com/). **d** Analysis using Liu_2014 data (*n* = 125) [40]. **e** Analysis using Tang_2018 data (*n* = 65) [41]

A total 83 BCA patients with tissue and serum specimens and 60 normal controls were used in this study. Tissues were stained with immunohistochemistry for PN and grading. Then the correlations between serum or tissue PN and clinical data were performed. Serum PN between normal controls and BCA patients showed no significant difference in medians (*P*-value = 0.511) (Fig. 6a) but were different in maximal values (130 ng/ml for normal control versus 194 ng/ml for BCA patients). By setting cut-point values of high levels of serum PN as higher than 94 ng/ml [14] and immunohistochemistry staining scores of 6–9 as high expression, the correlation between high and low expression of PN in tissue and serum by Fisher’s exact test showed significant differences with *P*-value equal to 0.010 (Table 1). Tissue PN staining was presented only in fibroblasts but not in cancer cells (Fig. 6b-c). Tissue PN expression showed a statistical significance only in correlation with presentation of ductal carcinoma in situ (DCIS) in pathological findings with a *P*-value equal to 0.039 (Table 2). By focusing the usage of anthracycline (doxorubicin or epirubicin) in duration of treatment, the success and failure of chemotherapeutic treatment were determined by recurrence or metastasis within 5 years after the course of anthracycline treatment. There were 32 cases included and only 2 cases that fitted criteria of failure (both had metastasis) and the statistical test was not significant (Table 2). The other correlation tests between expression of PN in either tissue or serum show no statistical significance with any of clinical data (Table 2). Kaplan-Meier Log-rank test from 2 sets of data (Liu_2014: total *n* = 126, selected for PN = 125 and Tang_2018: total *n* = 118, selected for PN = 65) showed significance correlation between PN protein expression and poor survival of BCA patients with 10-year analysis (Fig. 6d, e) [40, 41].

**Table 1:** Correlation between tissue PN expression and serum PN level in BCA patients (*P*-value = 0.010)

		Tissue PN (Q-score)		Total
		Score < 6	Score ≥ 6	
Serum PN (ng/ml)	≤ 94 ng/ml	51 (61.4%)	23 (27.8%)	74 (89.2%)
	> 94 ng/ml	2 (2.4%)	7 (8.4%)	9 (10.8%)
Total		53 (63.8%)	30 (36.2%)	83 (100%)

**Table 2:** Correlation between clinical presentation and tissue PN expression or serum PN level in BCA patients

Parameter	Condition	Tissue PN (Score)		***P***-value	Serum PN (ng/ml)		***P***-value
		< 6	≥ 6		≤ 94	> 94	
Age (Y)	≤ 50	27	14	0.442	37	4	1
	> 50	26	16		37	5	
BCA family history	Absence	46	25	0.449	64	7	0.612
	Presence	7	5		10	2	
Multi-loci	Absence	16	10	0.879	22	4	0.514
	Presence	36	20		51	5	
	Not determine	1	0		1	0	
DCIS	Absence	24	7	0.039*	27	4	0.722
	Presence	29	23		47	5	
Staging	0	6	9	0.166	13	2	0.389
	1	15	9		21	3	
	2	25	10		33	2	
	3	7	2		7	2	
Tumor length (mm)	< 20	20	10	0.291	27	3	1
	≥ 20	32	17		43	6	
	Not determine	1	3		4	0	
Lymph node	Absence	26	17	0.620	37	6	0.513
	Presence	12	4		14	2	
	Not determine	15	9		23	1	
Perineural invasion	Absence	19	12	0.177	26	5	0.534
	Presence	6	0		6	0	
	Not determine	28	18		42	4	
ER	Absence	14	4	0.361	17	1	0.742
	Presence	38	25		55	8	
	Not determine	1	1		2	0	
PR	Absence	16	7	0.819	20	3	0.769
	Presence	36	22		52	6	
	Not determine	1	1		2	0	
Clinical response to anthracycline	Bad	2	0	0.379	2	0	0.778
	Good	21	9		26	4	
	No anthracycline	30	21		46	5	

## Discussion

The latest Global Health Observatory data from the World Health Organization in 2016 showed that BCA is the 8th cause of death of females worldwide [42]. Even though there are many ways for early detection of BCA, many patients need chemotherapy. Doxorubicin or adriamycin is one of the first line chemotherapeutic agents in BCA patients [43]. It is a member of the anthracycline family of chemotherapeutic agents. It can intercalate double stranded DNA and inhibit topoisomerase II enzyme activity that then suppresses DNA replication and causes cytotoxicity [43]. Similar to almost cytotoxic drugs, doxorubicin also has serious adverse effects, especially immunosuppression and cardiotoxicity that can cause mortality in patients [44]. To overcome these adverse effects, nanoparticle formulation was introduced in the clinical use of doxorubicin [43], however, the cost of these formulas was much increased too.

This study successfully generated PN expression BCA cell lines and checked the expression of the PN receptor, integrins. All cells presented integrins expression especially αVβ5 heterodimer, which can act as PN receptor [3, 31], similar to the previous article for both commercial cell lines, MDA-MB-231 and MCF-7 [45]. This proved that these cells can respond to PN in an autocrine-paracrine manner. PN-transfected cells were used to screen for drug sensitivity. These results determined that only doxorubicin resistance was present in the current system. Doxorubicin has been reported in resistance in MDA-MB-231 and MCF-7 cell lines by in vitro experiments that showed resistance was mediated in the extracellular matrix [46]. Moreover, higher PN expression was presented in induced doxorubicin resistant W1 ovarian cancer cell lines compared with the parental cells [20]. This evidence can confirm present results that PN can induce doxorubicin in cancer cells. In addition, PN showed promotion of proliferation and migration in all BCA cells (Fig. 2), that corresponded to previous studies reported in CCA cells [9]. To reverse these effects, anti-PN peptide was designed to counteract PN on cancer promotion activities.

Only one anti-PN peptide against the integrin binding site was screened from the bacteriophage library. It showed affinity binding to PN, similar to commercial anti-PN antibody (Fig. 1). Physical properties of this peptide show stability in extracellular environment which is the location of secretory PN (Fig. S4). Peptide structure was showed as linear with small alpha helix (Fig. S5a) and bind to PN at active site (Fig. S5b). It could also inhibit PN-induced cell proliferation and migration in BCA cells, with minimal effect on cells without PN (Fig. 2). This evidence confirmed that the inhibitory effects were from binding to PN but not directly to the cells. The inhibition of PN-induced proliferation was shown better in rPN treatment than in the PN-transfected experiment; this may be that prolonged exposure of PN had more effect than short time treatment in the proliferation assay. For reversing of the doxorubicin resistance induced by PN, anti-PN peptide shifted the dose response curve to the left in PN presenting conditions (Fig. 3). This result indicated the effect of anti-PN peptide to improve drug resistance in BCA with PN expression. The results of the present study also demonstrated that the intracellular signaling mechanism might be via the phosphorylation of Akt and downstream expression of survivin (Fig. 4) as in previous reports [17, 18], therefore, this may also be considered as a target of repression of PN action in cancer promotion. Stemness has been recognized as a molecular mechanism of chemoresistance including in breast cancer [47]. This report also determined survivin as an upstream molecule of stemness. In addition, PN has been also recognized as a cancer stemness marker too [48]. Our results showed higher CD24^−^/CD44^+^ stem cell in PN-transfected MCF-7 compared with mock control and it could be reduced by anti-PN peptide. MDA-MB-231 BCA cell has also shown the similar pattern but less different than MCF-7 according to the baseline of stemness in MDA-MB-231 was much higher than MCF-7. Unfortunately, the statistical test was not significant according to minimally change in MDA-MB-231 cell and high error bar in MCF-7 cell. A further study might be in an in vivo system toward implementation for clinical usage. Since anti-PN antibody was studied to inhibit PN-promoted cancer progression in mouse model [49], anti-PN peptide should have an advantage providing more ability for tissue penetration [50].

In proliferation experiments, anti-PN peptide showed inhibition only in PN treatment but seemed not in PN-transfected experiment. The explanation might start that PN actions as paracrine in almost studies [3, 4]. However, some reports showed that some cells such as keratinocytes, renal mesangial, renal tubular epithelial cells and BCA cells expressed PN but not secreted [51]. Moreover, few studies indicated the localization of PN in cytoplasm and nucleus of BCA (MCF-7 and MDA-MB-468) and COS7 cells [52, 53]. In addition, some cytokines showed the function in both extracellular and intracellular, such as IL33 and HMGB1, for examples [54, 55]. A report showed that overexpression renal mesangial cell had increasing of proliferation and fibronectin secretion without evidence of extracellular PN [51]. The other study indicated the knockdown of either integrin αVβ3 or PN in lung cancer cell could reduce cell proliferation and double knockdown showed much lower [56]. These phenomena might be the explanation of intracellular PN function on cell proliferation that anti-PN peptide could not or minimally inhibit the cell proliferation in BCA cells. BCA cells including MCF-7 and MDA-MB-231 had been reported the minimal expression of PN [57]. In this study, the overexpressed PN could be secreted and detected in all PN-transfected BCA cells. In addition, the intracellular signaling via pAKT and survivin could be inhibited by anti-PN peptide. However, if the transfection of PN plasmid which was not the natural condition could introduce some intracellular function of PN, therefore, anti-PN peptide could not inhibit this mechanism. This phenomenon was showed only in proliferation but not migration assay which might have different regulation. To answer this question, the further experiments such as manipulation of PN secretion by inhibitor and examine the proliferation/migration activity should be performed.

Eighty-three BCA patients with tissue and serum specimens were included in this study. Immunohistochemistry in this study’s setting did not show staining of PN in cancer cells, neither in the previous studies in CCA [9, 14], while signals in the stroma area were very strong (Fig. 5). In addition, the results from real time RT-PCR of both BCA cell lines showed high baselines of Ct values (approximately 32 and 39 for MDA-MB-231 and MCF-7, data not shown), implying that the expression was low. For MCF-7, the Ct value was equal to a previous report [52]. Immunohistochemical results, however, did not correlate with the previous reports [52, 57]. The variation might be from the antibody, staining conditions, background and also different sample groups. The results were confirmed for only stromal staining without being positive in cancer cells. The results showed that tissue PN expression and serum PN level had a significant correlation, however, only 36.2% of cases had strong positive of PN staining and only 10.8% had a high level of serum PN. The median of serum PN levels in BCA patients did not differ from normal controls, but the maximum was higher. Analysis by online database showed significant correlation between PN protein expression and poor survival of BCA patients (Fig. 6d, e), similar to recent publication [16]. However, in this study, there was no correlation between serum PN and clinical data. This finding supported a previous study in early BCA [15]. While the other report showed opposite [57]. The explanation might be that the expression of PN in that publication was in cancer cells but the present result was in stroma cells, so that the results could be different. Since prognosis of treatment in this group of BCA patients was good, there were only 32 patients (38.6%) received an anthracycline (doxorubicin or epirubicin), so it may be a lot of parameters that could interfere the anthracycline-based treatment. Taken together, this indicated that PN seemed not to be significant in the all-over BCA picture but might be important in some situations. Unlike CCA [9, 14], PN have no clinical significance in BCA may because of the small size of the cancer tissue, compared with CCA.

### Conclusions

This study established a new anti-PN peptide that could counteract PN-mediated cancer progression including induced doxorubicin resistance. This peptide could be developed for clinical usage in the future, not only for BCA, but also for other cancers such as CCA.

## Supplementary Information

**Additional file 1: Figure S1.** Detection of intergrin α5, α6, αV, β1, β3, β4 and β5 mRNA expression in BCA cell lines by real time RT-PCR. Error bar determined SEM.

**Additional file 2: Figure S2.** The expression of intergrin αVβ5 in BCA cell lines by immunofluorescent staining. All cells showed positive staining with membrane pattern (red signal) and blue nucleus from Hoechst 33258. These pictures were taken from confocal microscope with 640X original magnification. (a) MDA-MB-231 and (b) MCF-7.

**Additional file 3: Figure S3**. Detection of PN in PN- and mock transfected BCA cell lines. (a) Detection of mRNA expression by real time RT-PCR and (b) Detection of PN protein in condition medium by western blot analysis. All cells showed increasing of PN signal in PN-transfected condition compared to mock cells by adjusting equal protein loading. M = mock transfected and PN = PN-transfected, error bar determined SEM.

**Additional file 4: Figure S4.** Physical properties of the TFATHGKHWAAP peptide. The analysis was performed by online tool (https://www.thermofisher.com). Net charge at pH 7.4 is approximately 1 (red arrow in graph).

**Additional file 5: Figure S5.** Prediction of TFATHGKHWAAP peptide structure and binding to PN. The analysis was performed by RPBS online tools (https://bioserv.rpbs.univ-paris-diderot.fr). (a) Prediction of peptide structure by PEPFOLD3 protein structure prediction tool. The structure is almost linear with small α-helix at N-terminal. (b) Prediction of binding between peptide (red) and PN protein (blue). The binding site is located near the active site (yellow area) of PN. The binding energy of this model was -11.89 kCal/mol.

**Additional file 6: Figure S6.** Drug response study was determined by IC50 of cells. The comparison was performed between mock and PN-transfected BCA cells. (a) Doxorubicin, (b) Cisplatin, (c) Paclitaxel, (d) transfected, error determined SEM, * = *P*-value< 0.05 and n.s. = not significant.

**Additional file 7: Table S1.** Primers for real time RT-PCR.

## Data Availability

The dataset used in the current study are available from corresponding author according to the reasonable request.

## Abbreviations

PN: Periostin
BCA: Breast cancer
TME: Tumor microenvironment
NSCLC: Non-small cell lung cancer
CCA: Cholangiocarcinoma
CAF: Cancer associated fibroblast
CRC: Colorectal cancer
HCC: Hepatocellular carcinoma
OVC: Ovarian cancer
5-FU: 5-fluorouracil
US-FDA: United States Food and Drug Administration
DMEM/F-12: Dulbecco Modified Eagle’s Medium/F12
DMEM: Dulbecco Modified Eagle’s Medium
FBS: Fetal Bovine Serum
RT: Reverse transcriptase
PCR: Polymerase chain reaction
GAPDH: Glyceraldehyde-3-phosphate dehydrogenase
Ct: Cycle threshold
PBS: Phosphate buffered saline
SDS: Sodium dodecyl sulfate
PAGE: Polyacrylamide gel electrophoresis
BSA: Bovine serum albumin
HRP: Horse reddish peroxidase
ECL: Enhanced chemiluminescence
FITC: Fluorescein isothiocyanate
MTS assay: CellTiter 96® Aqueous One Solution Cell Proliferation Assay
