# Supplementary figures and images for: Development of an engineered peptide antagonist against periostin to overcome doxorubicin resistance in breast cancer
Source: BMC Cancer. 2021 Jan 14;21:65. doi: 10.1186/s12885-020-07761-w (PMC7807878; doi:10.1186/s12885-020-07761-w)

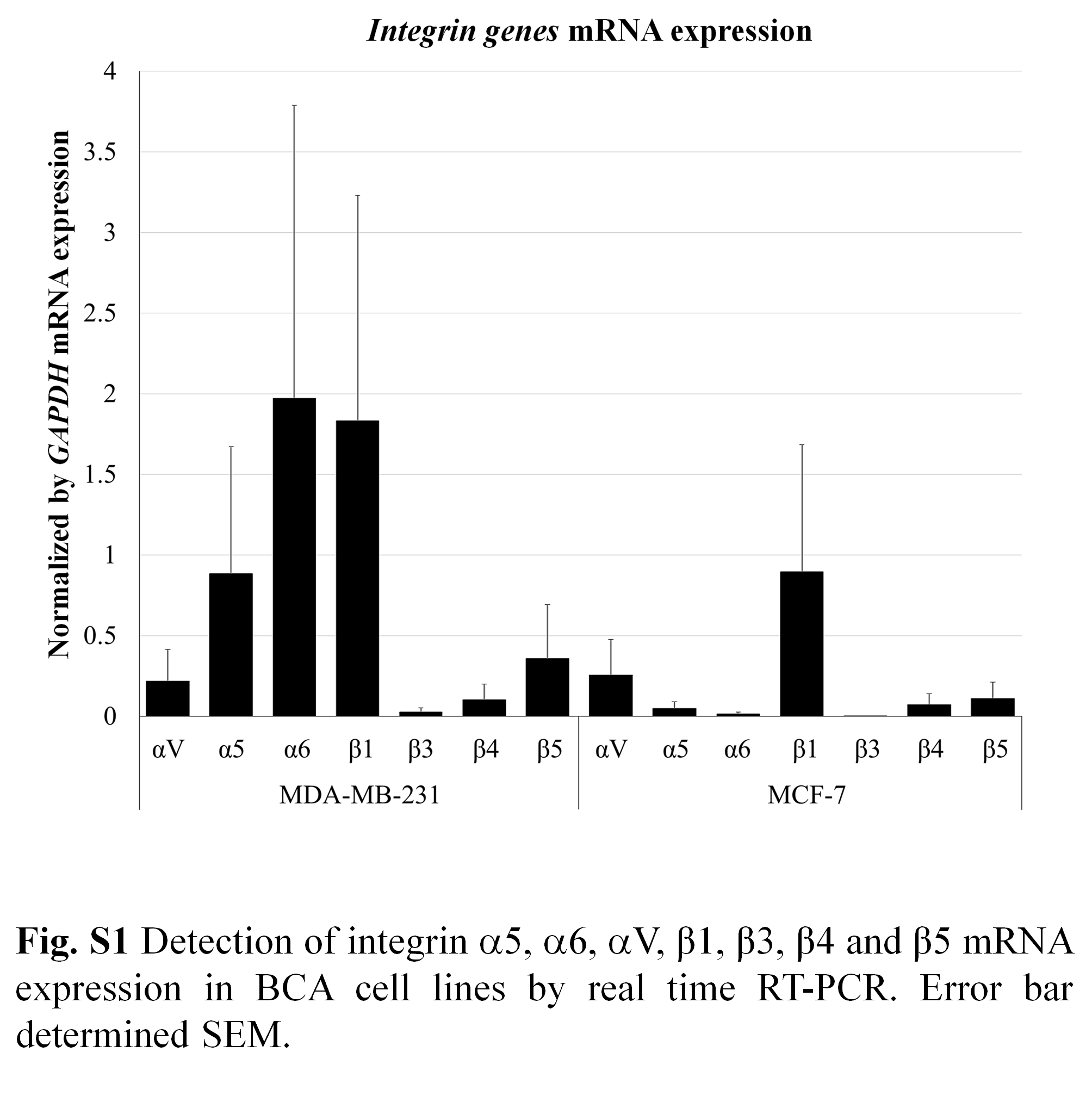

Supplement: Supplementary file 1 — Additional file 1: Figure S1. Detection of intergrin α5, α6, αV, β1, β3, β4 and β5 mRNA expression in BCA cell lines by real time RT-PCR. Error bar determined SEM. [file 12885_2020_7761_MOESM1_ESM.tif]

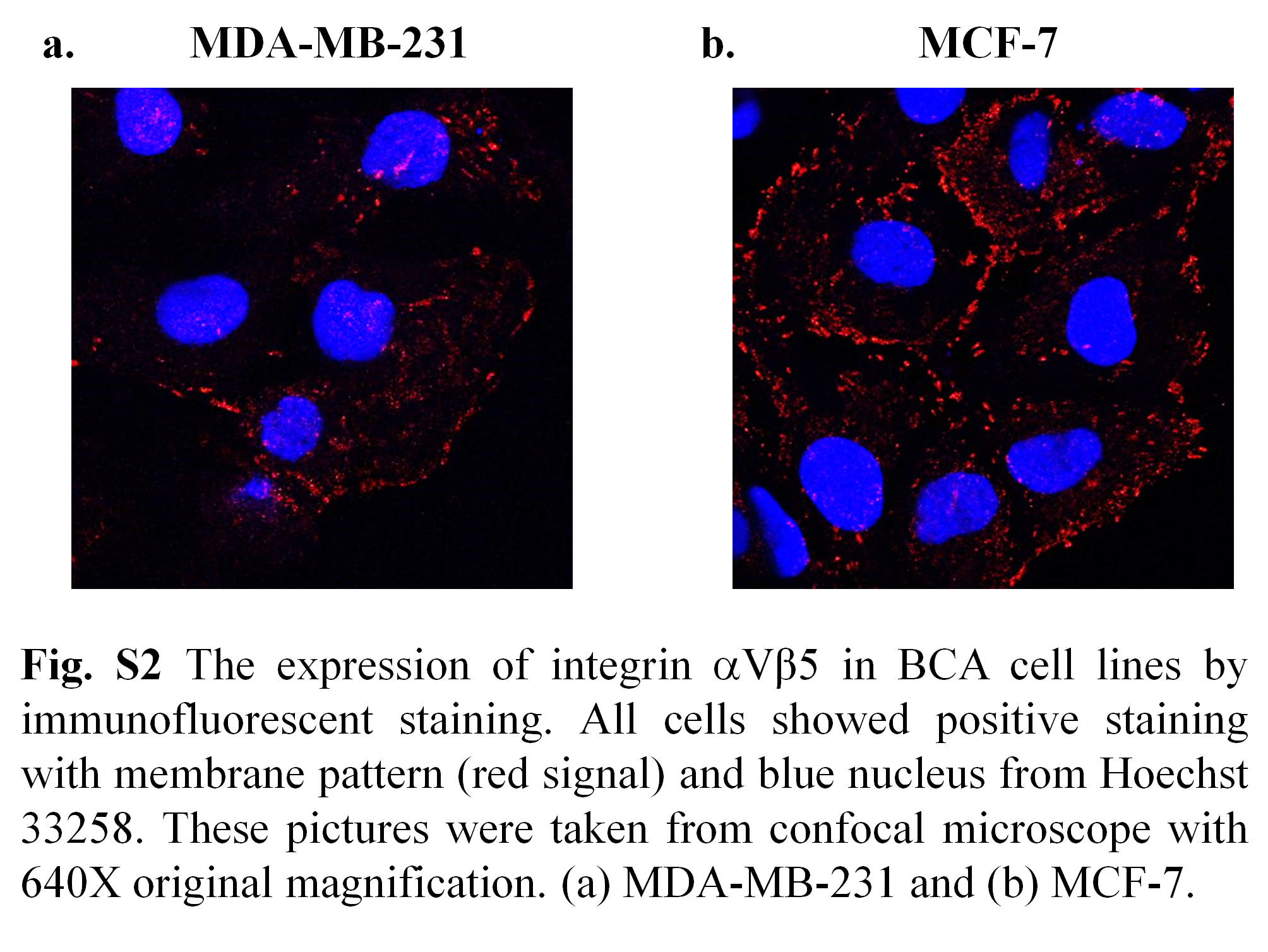

Supplement: Supplementary file 2 — Additional file 2: Figure S2. The expression of intergrin αVβ5 in BCA cell lines by immunofluorescent staining. All cells showed positive staining with membrane pattern (red signal) and blue nucleus from Hoechst 33258. These pictures were taken from confocal microscope with 640X original magnification. (a) MDA-MB-231 and (b) MCF-7. [file 12885_2020_7761_MOESM2_ESM.tif]

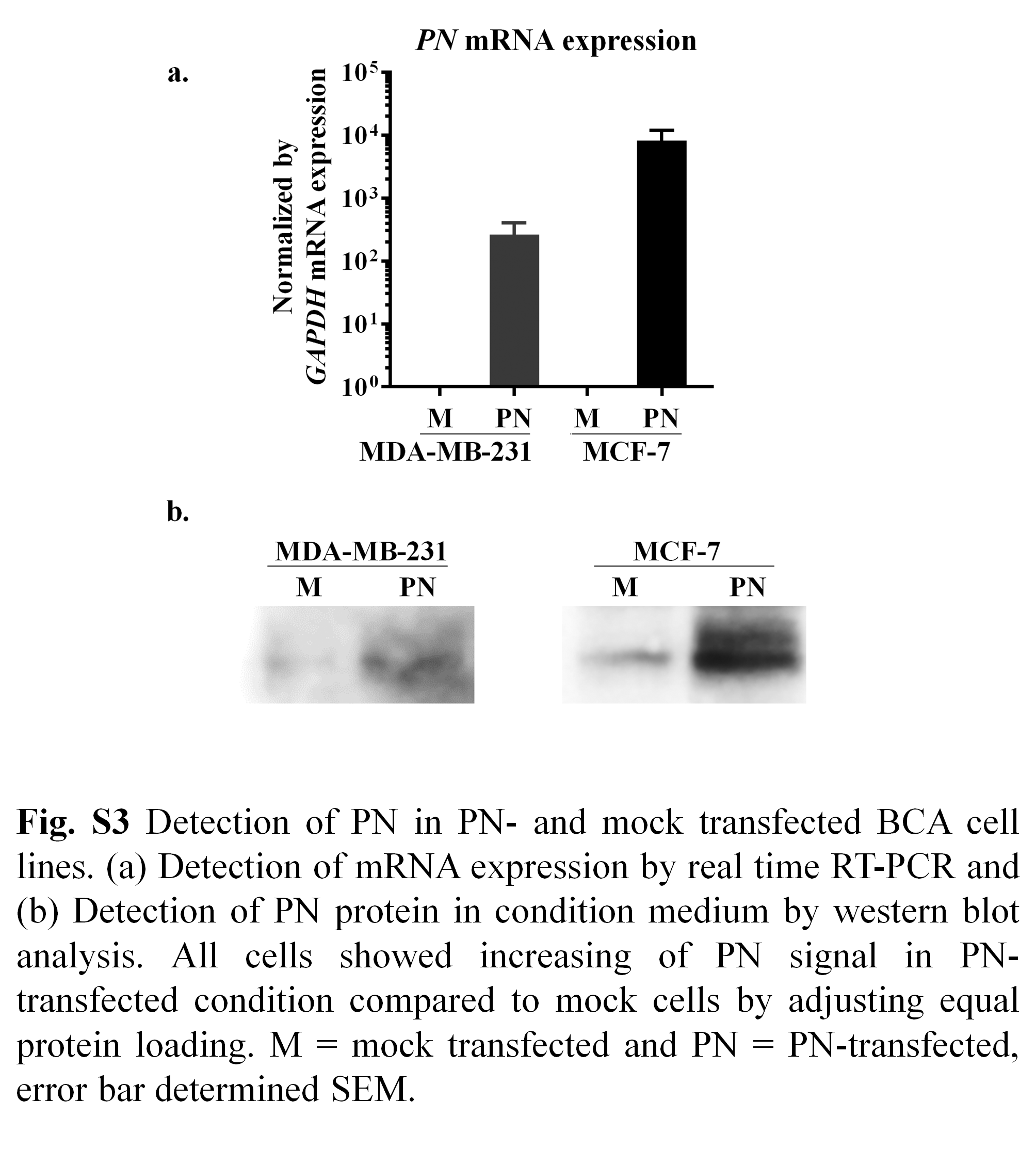

Supplement: Supplementary file 3 — Additional file 3: Figure S3. Detection of PN in PN- and mock transfected BCA cell lines. (a) Detection of mRNA expression by real time RT-PCR and (b) Detection of PN protein in condition medium by western blot analysis. All cells showed increasing of PN signal in PN-transfected condition compared to mock cells by adjusting equal protein loading. M = mock transfected and PN = PN-transfected, error bar determined SEM. [file 12885_2020_7761_MOESM3_ESM.tif]

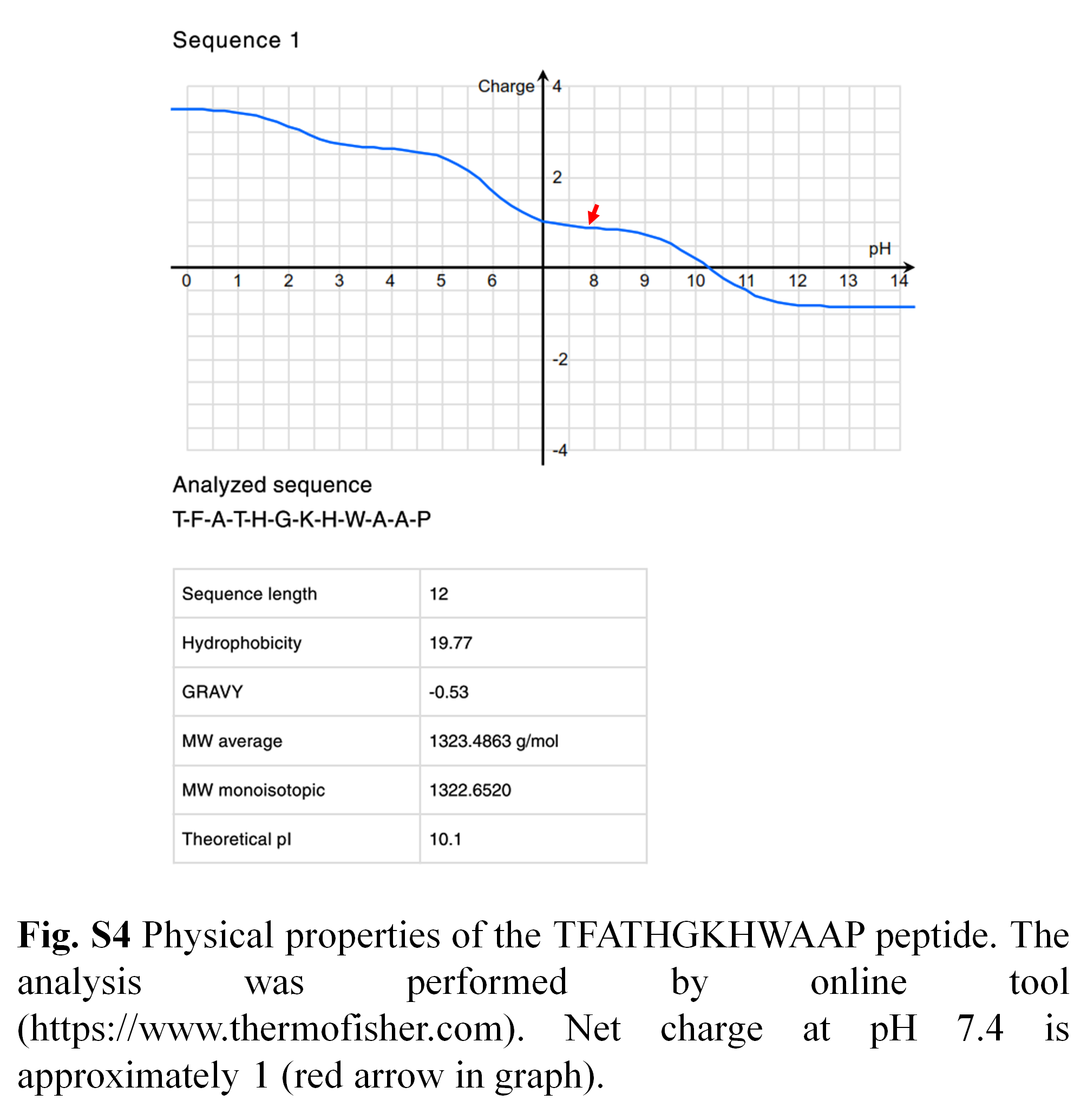

Supplement: Supplementary file 4 — Additional file 4: Figure S4. Physical properties of the TFATHGKHWAAP peptide. The analysis was performed by online tool (https://www.thermofisher.com). Net charge at pH 7.4 is approximately 1 (red arrow in graph). [file 12885_2020_7761_MOESM4_ESM.tif]

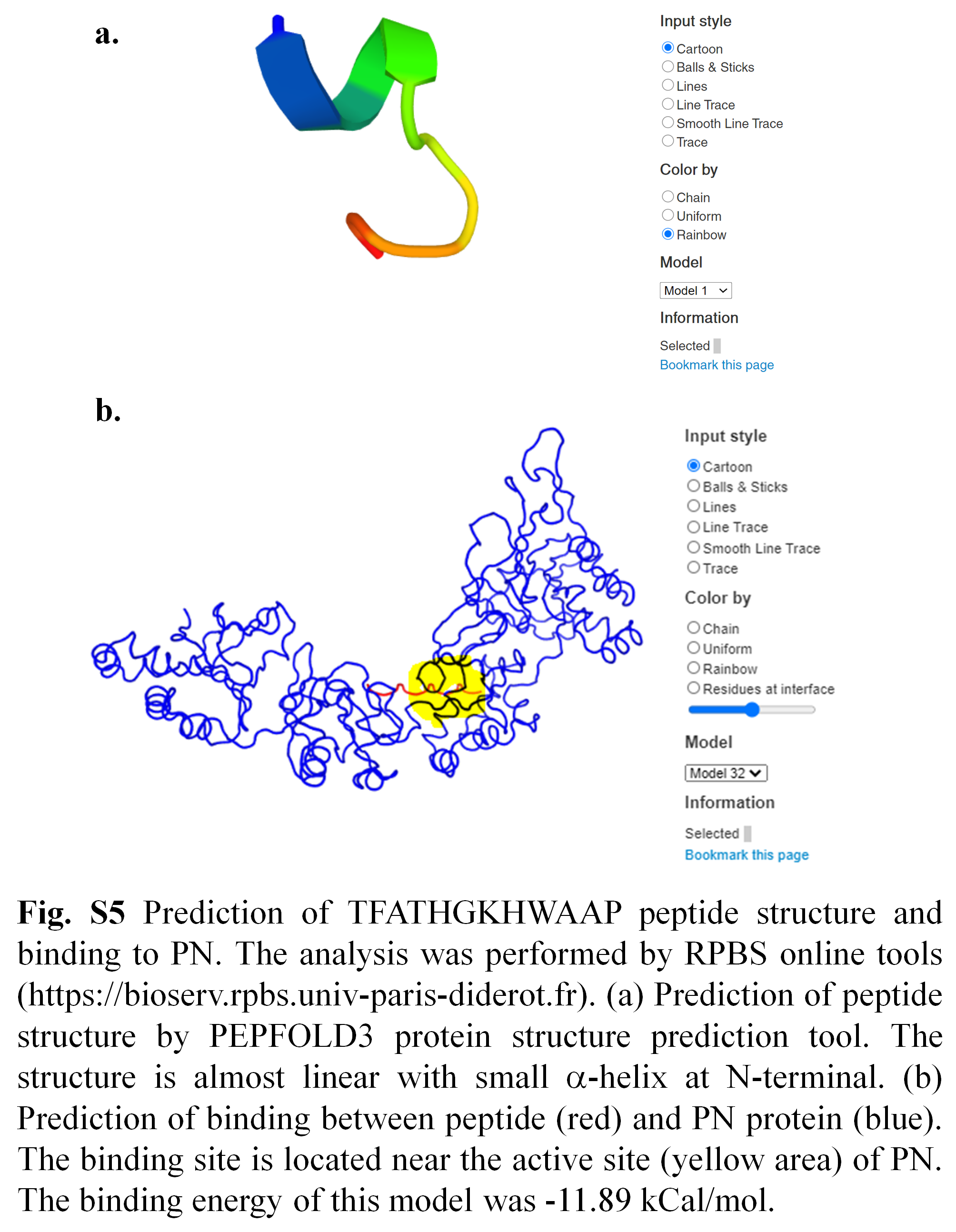

Supplement: Supplementary file 5 — Additional file 5: Figure S5. Prediction of TFATHGKHWAAP peptide structure and binding to PN. The analysis was performed by RPBS online tools (https://bioserv.rpbs.univ-paris-diderot.fr). (a) Prediction of peptide structure by PEPFOLD3 protein structure prediction tool. The structure is almost linear with small α-helix at N-terminal. (b) Prediction of binding between peptide (red) and PN protein (blue). The binding site is located near the active site (yellow area) of PN. The binding energy of this model was -11.89 kCal/mol. [file 12885_2020_7761_MOESM5_ESM.tif]

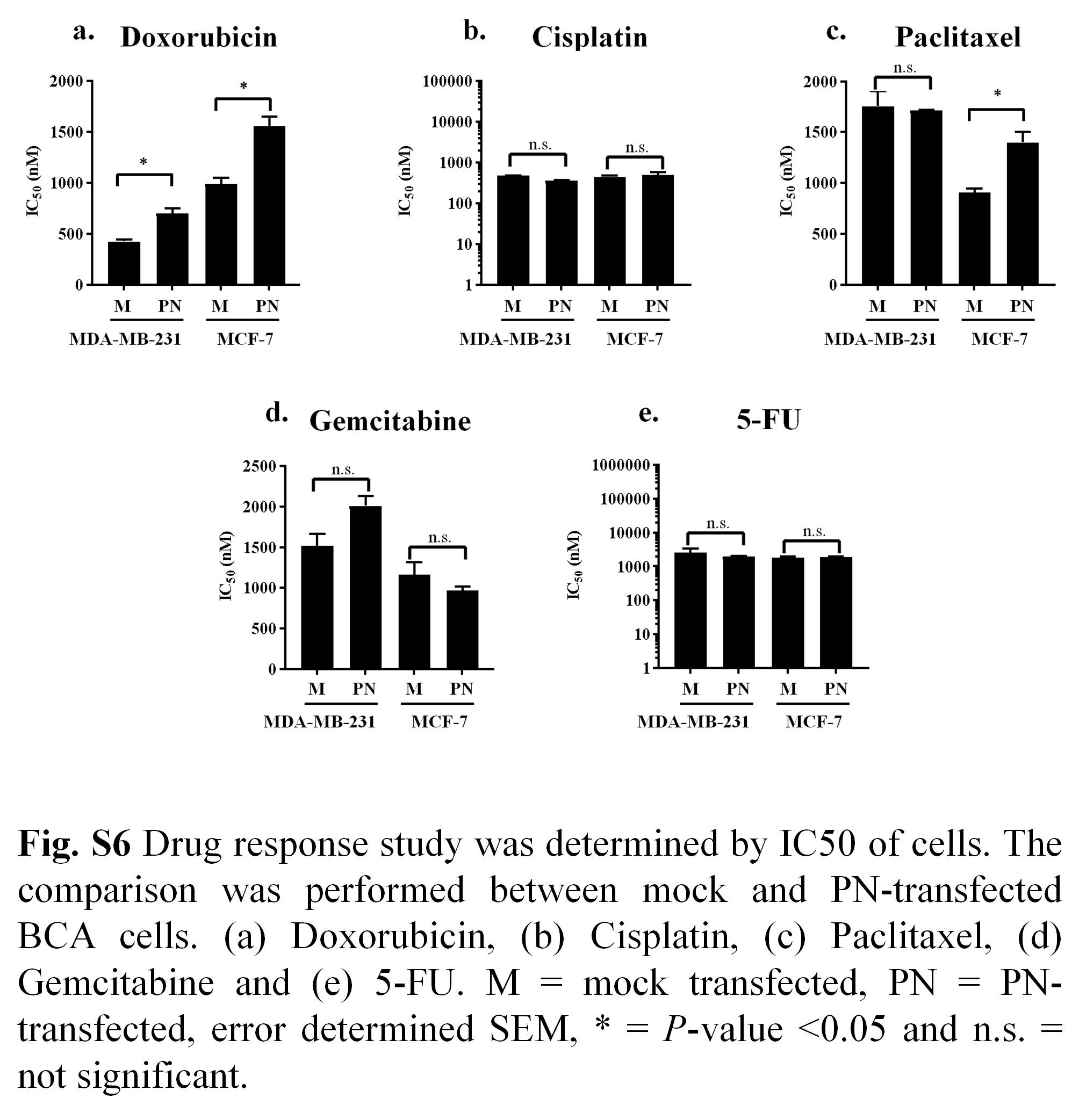

Supplement: Supplementary file 6 — Additional file 6: Figure S6. Drug response study was determined by IC50 of cells. The comparison was performed between mock and PN-transfected BCA cells. (a) Doxorubicin, (b) Cisplatin, (c) Paclitaxel, (d) transfected, error determined SEM, * = P-value< 0.05 and n.s. = not significant. [file 12885_2020_7761_MOESM6_ESM.tif]
