# Supplementary material for: Development of an engineered peptide antagonist against periostin to overcome doxorubicin resistance in breast cancer
Source: BMC Cancer. 2021 Jan 14;21:65. doi: 10.1186/s12885-020-07761-w (PMC7807878; doi:10.1186/s12885-020-07761-w)
Supplement: Supplementary file 7 — Additional file 7: Table S1. Primers for real time RT-PCR. [file 12885_2020_7761_MOESM7_ESM.docx]

**Additional file 7: Table S1.** Primers for real time RT-PCR

| **Gene** | **Forward primer** | **Reverse primer** | **Size (bp)** |
| --- | --- | --- | --- |
| *PN* | CACTCTTTGCTCCCACCAAT | TCAAAGACTGCTCCTCCCATA | 157 |
| *GAPDH* | CGAGATCCCTCCAAAATCAA | TTCACACCCATGACGAACAT | 170 |
| *ITGA5* | AGTTGCATTTCCGAGTCTGG | CCAAACAGGATGGCTAGGAT | 223 |
| *ITGA6* | GGCCTTATGAAGTTGGTGGA | CTCTGGGAGCACCAGATACAA | 144 |
| *ITGAV* | GTGACTGGTCTTCTACCCGC | CTCACAGATGCTCCAAACCA | 121 |
| *ITGB1* | TCCCTGAAAGTCCCAAGTGT | TTTCCTGCAGTAAGCATCCA | 143 |
| *ITGB3* | TGGTCCTGCTCTCAGTGATG | TGAAGGTAGACGTGGCCTCT | 180 |
| *ITGB4* | TCTCCTACCGCACACAGGA | CTTCACCTGCAGCTCTTTCC | 110 |
| *ITGB5* | CTCCACTCTGGGAAACCTGA | AGGACGGTCAGGTTGGACTT | 188 |
